# Social Influence Maximization in Hypergraphs †

**DOI:** 10.3390/e23070796

**Published:** 2021-06-23

**Authors:** Alessia Antelmi, Gennaro Cordasco, Carmine Spagnuolo, Przemysław Szufel

**Affiliations:** 1 Dipartimento di Informatica, Università degli Studi di Salerno, 84084 Fisciano, Italy; 2 Dipartimento di Psicologia, Università degli Studi della Campania “Luigi Vanvitelli”, 81100 Caserta, Italy; gennaro.cordasco@unicampania.it; 3 Decision Analysis and Support Unit, SGH Warsaw School of Economics, 02-554 Warsaw, Poland; pszufe@sgh.waw.pl

**Keywords:** hypergraphs, high-order networks, influence diffusion, target set selection, social networks

## Abstract

This work deals with a generalization of the minimum Target Set Selection (TSS) problem, a key algorithmic question in information diffusion research due to its potential commercial value. Firstly proposed by Kempe et al., the TSS problem is based on a linear threshold diffusion model defined on an input graph with node thresholds, quantifying the hardness to influence each node. The goal is to find the smaller set of items that can influence the whole network according to the diffusion model defined. This study generalizes the TSS problem on networks characterized by many-to-many relationships modeled via hypergraphs. Specifically, we introduce a linear threshold diffusion process on such structures, which evolves as follows. Let H=(V,E) be a hypergraph. At the beginning of the process, the nodes in a given set S⊆V are influenced. Then, at each iteration, (i) the influenced hyperedges set is augmented by all edges having a sufficiently large number of influenced nodes; (ii) consequently, the set of influenced nodes is enlarged by all the nodes having a sufficiently large number of already influenced hyperedges. The process ends when no new nodes can be influenced. Exploiting this diffusion model, we define the minimum Target Set Selection problem on hypergraphs (TSSH). Being the problem NP-hard (as it generalizes the TSS problem), we introduce four heuristics and provide an extensive evaluation on real-world networks.

## 1. Introduction

Social influence involves intentional and unintentional efforts to change another person’s beliefs, attitudes, or behavior [1]. With the advent of online social networks, such a phenomenon assumed even more importance given the exponential number of people a user can reach through them. In both online and offline contexts, a person may influence other individuals for different reasons. As a domino cascade, such influence impacts not only those people but also propagates through the social network, with varying degrees of effectiveness. This *word-of-mouth* propagation has been shown to be a powerful tool in different applications, such as viral marketing [2,3], recommendation [4,5,6], influential bloggers identification [7,8,9] and expert finding [10,11].

Social influence occurs due to the diffusion of information in the network [12]. A key algorithmic question in information diffusion research is social influence maximization (SIM), a well-studied problem due to its potential commercial value [13]. SIM aims to select a seed set of *k* users in an online social network, abstracted as a graph, such that the expected number of influenced users through the seed set is maximized. Spreading phenomena are usually modeled as diffusion processes within graphs. As a trivial consequence, problems like SIM and its variants are analyzed via such structures. However, graphs assume the existence of only binary relationships between nodes, even though more articulated interactions characterize real-world networks as interactions between the elements may not be dyadic but may involve more than two entities. Examples of such scenarios include membership in groups on social platforms, co-authorships of scientific publications, or several parties participating in a crypto-currency transaction [14]. In such cases, nodes may be linked together based either on explicit information (e.g., inclusion in groups) or implicit information (e.g., whether online social network users share the same hashtag in a media post or review the same restaurant). A natural way to represent such many-to-many relations among entities without loss of information are hypergraphs [15]. Being a generalization of graphs, where a (hyper)edge is a relationship among an arbitrary number of nodes, such mathematical structures can naturally define higher-order relations between groups of objects. Hence, hypergraphs enable us to account for complex dynamics happening in real life, like the influence exerted by a *group* of friends [16].

Based on the above considerations and motivated by the lack of comprehensive studies towards this direction, we analyze the SIM problem on social networks abstracted as hypergraphs. In our previous work [17], we formally defined a social influence diffusion process on hypergraphs, and we presented a variant of the minimum target set selection problem, first proposed by Kempe et al. [18], suitable for networks involving many-to-many relationships. We developed three greedy-based heuristics to address such a problem, whose aim is to select the smallest set of initially influenced users such that, at the end of the influence diffusion process, all the users are influenced. We evaluated and compared the proposed algorithms on a set of synthetic hypergraphs, varying the networks’ properties, and on a real use-case, based on the network induced by the co-occurrences of characters in the Game-of-Thrones TV Series.

This paper is an extension of the previous work just described [17], and its major contributions can be summarized as follows:

- We revise the three greedy approaches proposed in [17], adding an optimization phase that removes all the unnecessary nodes untimely added by the greedy procedure.
- We design a new greedy algorithm based on a subtractive approach, inspired by Cordasco et al. [19], which iteratively prunes non-influential nodes or edges from the hypergraph until some nodes become essential to the diffusion process.
- We extensively evaluate all the proposed heuristics on real-world networks.

The remainder of the paper is organized as follows. Section 2 reviews some relevant literature about the social influence problem, specifically focusing on higher-order networks. In Section 3, we define a diffusion process on hypergraphs and the problem of finding a minimum target set for those structures. Section 4 describes the proposed greedy-based heuristics to solve the target set selection problem on hypergraphs. In Section 5, we present the experiments on real-world networks and discuss their outcomes. Finally, Section 6 details the conclusion and future work.

## 2. Related Work

### 2.1. The Social Influence Maximization Problem in Graphs

Initially introduced by Domingos and Richardson in the context of viral marketing [20], the Social Influence Maximization (SIM) problem has been studied since early two thousand. Three recent surveys give a thorough description of the problem and its variants, solution methodologies of the problem, and real-life applications [12,13,21].

Kempe et al. [18] first modeled influence maximization as an algorithmic problem in 2003. The problem studies a social network abstracted as a graph G=(V,E), where *V* is the set of nodes (i.e., users) and *E* is the set of (directed/undirected) edges (i.e., social links between users). The goal is to find a *k*-sized set of users with the maximum influence in *G*. In its basic version, the Target Set Selection (TSS) problem [22] adds another parameter to the SIM problem, asking to find out a subset of at most *k* nodes, such that after the diffusion process over at least λ nodes are influenced. In this work, we consider a variant of the TSS problem where we aim to find a minimum target set that will eventually influence the whole network under a linear threshold (LT) influence propagation model. According to the LT model, introduced by Granovetter [23] and Schelling [24] in 1978, a user *v* becomes active when the sum of influences of its neighbors in the networks reaches a specific threshold t(v).

Kempe et al. [18] were also the first to investigate the computational complexity of the SIM problem, proving it to be NP-hard to approximate within a factor of $O(n^{\left(1-\epsilon \right)})$ for all ϵ>0. While Kempe et al. [18] analyzed the problem in networks with randomly chosen thresholds, Chen [25] studied the minimization problem of finding the smallest target set able to influence the whole network built with fixed arbitrary thresholds. Furthermore, Chen proved a strong inapproximability result that makes unlikely the existence of an algorithm for the TSS problem on graphs (2-uniform hypergraphs) with an approximation factor better than $O(2^{\log^{1-\epsilon }\left|V\right|})$. Cordasco et al. [19] presented an algorithm for the TSS problems on graphs, which provides an optimal solution (i.e., a minimum size subset of nodes that influence the whole network) in case the network is either a tree, a cycle, or a complete graph.

Another line of inquiry in the context of social influence diffusion is understanding how diffusion mechanics differ across social media as well as modeling different types of influence within a network. Kim et al. [26] moved towards the first direction, exploring news diffusion instances across different social media platforms and finding that influence between different media types is varied by the context of information. Li et al. [27] followed the second direction, formally defining a multiple influences diffusion model by considering the influential relationships and individual’s personalized traits, such as interests and trusts. Recently, Chathurani et al. [28] put together these two aspects, investigating several influence patterns exercised by different users within and across online social media platforms.

### 2.2. The Social Influence Maximization Problem in Higher-Order Networks

Little or no literature exists on the SIM problem on hypergraphs as these structures rose to prominence only recently in the academic and industry landscape.

In 2019, Zhu et al. [16] first dealt with the study of information diffusion in social networks abstracted as a directed hypergraph. They model the crowd influence as a hyperedge $e=(H_{e},v)$ with weight $0\leq P_{e}\leq 1$, where $H_{e}$ is the head node-set and *v* is the tail node, meaning that *v* will be influenced by $H_{e}$ with probability $P_{e}$ only after each node in $H_{e}$ is influenced. Their proposed algorithm selects *k* initially-influenced seed users in a directed hypergraph G=(V,E,P), maximizing the expected number of eventually-influenced users. Zhu et al. prove the problem to be NP-hard under an Independent Cascade (IC) model [29] and propose an algorithm preserving a (1−1/e−ϵ)-approximation. In another work, the same authors revise the SIM problem under a group influence perspective [30]. Resembling real-life dynamics, they define the Group IM (GIM) problem. The problem aims to select *k* seed users such that the number of eventually influenced groups is maximized, where a group is influenced if at least a fixed percentage β of users in it are influenced. After analyzing the complexity and approximability of the GIM problem, showing that they are NP-hard, Zhu et al. propose an approximation framework for the problem. Differently from those two works, in our study, we specifically address the TSS problem under an LT diffusion model on networks characterized by many-to-many relationships and abstracted as undirected hypergraphs.

Influence diffusion models are usually treated as specific cases of epidemic models [21]. Ma and Guo [31] constructed and analyzed four kinds of information transmission patterns within the members of an enterprise under the epidemic transmission framework. In their model, a hyperedge represents an informal organization (e.g., spontaneous groups), and, according to the SIR model, nodes belong to the three standard classes corresponding to ignorant (S), spreader (I), and stifler nodes (R). In the probabilistic transmission model, all nodes are ignorant at the start. At the first iteration, the information begins to spread from a randomly chosen initial node to other randomly selected spreader nodes within the same hyperedge with a given probability. Immune nodes no longer spread the information and stop transmitting. The authors also analyzed variations of the model where information passes (i) from one person to another in a chain-like fashion (one-way transmission), (ii) to the entire hyperedge or group (gossip transmission), or (iii) to a constant number of nodes within the same hyperedge (group transmission).

A thorough description of epidemic spreading processes in higher-order networks, considering both simplicial complexes and hypergraphs, is given in a survey by Battiston et al. [14], in which the authors also discuss opinion and cultural dynamics beyond pairwise interactions, focusing on the Voter and Majority Models.

## 3. Social Influence Diffusion on Hypergraphs

In this section, after introducing hypergraphs and their graph representations briefly, we define an influence diffusion process on hypergraphs as a generalization of the LT model proposed by Kempe et al. in their seminal work on influence maximization [18]. We further describe the minimum target set selection problem on hypergraphs that we will discuss in Section 4.

### 3.1. Hypergraphs

A hypergraph is a generalization of a graph in which a hyperedge can join any number of nodes instead of only two entities, as happens for regular graphs. Formally, a hypergraph is an ordered pair H=(V,E), where *V* is a set of nodes, and *E* is the set of (hyper)edges ($E\subseteq 2^{V}\setminus \left\{\emptyset \right\}$). We denote with n=|V| the order of the hypergraph (i.e., the number of nodes) and with m=|E| the number of hyperedges. For each v∈V, we denote with E(v)⊆E the set of edges containing *v* and with d(v)=|E(v)| the degree of *v*. Similarly, for each he∈E, we denote with V(he)⊆V the set of nodes in he and with κ(he)=|V(he)| the cardinality of he.

**Figure 1:**
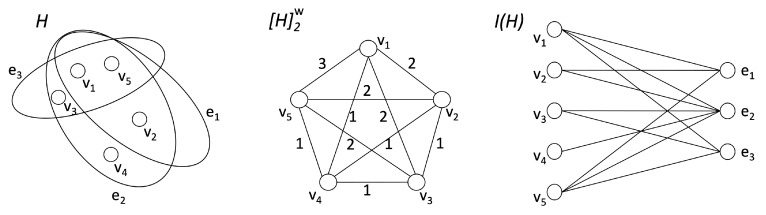
(**left**) A hypergraph H=(V,E) with five nodes and three hyperedges; (**center**) The weighted clique expansion ${\left[H\right]}_{2}^{w}$ associated with *H* (the edge weight, depicted on each edge, corresponds to the number of hyperedges containing both endpoints); (**right**) The bipartite incidence graph I(H) associated with *H* (the five nodes appear on the left and the three hyperedges appear on the right).

Several algorithms on hypergraphs are based on a graph representation of those structures. For instance, a two-section representation (or clique-expansion) of H=(V,E), denoted with ${\left[H\right]}_{2}=\left(V,E^{\prime }\right)$ and where $E^{\prime }=\left\{\left(u,v\right):u,v\in he,he\in E\right\}$, can be obtained by connecting two nodes in the graph ${\left[H\right]}_{2}$ if and only if they belong to the same hyperedge [32]. As a result, each hyperedge from *H* occurs as a complete subgraph in ${\left[H\right]}_{2}$. In this work, we consider the weighted clique-expansion ${\left[H\right]}_{2}^{w}=\left(V,E^{\prime },w\left(E^{\prime }\right)\to N\right)$ of *H*, where the weight of an edge e=(u,v) in ${\left[H\right]}_{2}^{w}$ corresponds to the number of hyperedges containing both *u* and *v*. A hypergraph H=(V,E) can also be considered as a bipartite incidence graph I(H)=(V∪E,F), where F={(v,he):v∈he,he∈E}. In other words, I(H) represents the bipartite network obtained from *H* by considering the nodes on one side and the hyperedges on the other side and where a node *v* is connected with a hyperedge he if and only if the node *v* is contained in the hyperedge he∈E. Figure 1 shows an example of a hypergraph and its corresponding weighted clique-expansion and incidence graph representations.

### 3.2. A Dynamic Social Influence Diffusion Process

Given a network represented with a hypergraph H=(V,E), we consider a dynamic diffusion process on *H* influencing both nodes and hyperedges. The idea behind the process is to model the diffusion iteratively from nodes to hyperedges and from hyperedges to nodes so that it is possible to manage non-binary relations (as hyperedges can have any size). Each neighbor of a node *v* can have a different impact on influencing *v* itself, depending on the number and the size of the hyperedges shared with the node. Modifying the thresholds associated with the hyperedges allows us to model different scenarios. The diffusion process evolves in discrete steps as described in [17].

In the following, we provide an easier description of the same process, noting that the incidence graph I(H) associated with a hypergraph *H* is an ordinary graph.

Let H=(V,E) be a hypergraph and I(H)=(V∪E,F) the associated incidence graph. Let $t_{V}:V\to N=\left\{0,1,\ldots \right\}$ and $t_{E}:E\to N=\left\{0,1,\ldots \right\}$ be two functions assigning thresholds to the nodes and to the hyperedges, respectively. For each node v∈V (he∈E), the value $t_{V}\left(v\right)$ ($t_{E}\left(he\right)$) quantifies how hard it is to influence the node *v* (edge he), in the sense that easy-to-influence elements of the network have *low* threshold values, and hard-to-influence elements have *high* threshold values. Let t(·) be a threshold function which assigns to each node of I(H) the same threshold value they have on *H*, defined as

$$t\left(v\right)=\left\{\begin{array}{c} t_{V}\left(v\right),\mathrm{if}v\in V\\ t_{E}\left(v\right),\mathrm{if}v\in E. \end{array}\right.$$

**Definition** **1.** *Let I(H)=(V∪E,F) be a incidence graph associated with a hypergraph H=(V,E) with threshold function t:V⟶N. An information diffusion process in I(H), starting with a seed S, is a sequence of nodes subsets*

I[S,0]⊆I[S,1]⊆…⊆I[S,ℓ]⊆…⊆V∪E,

*with I[S,0]=S, and such that for all ℓ>0,*

$$I\left[S,\ell \right]=I\left[S,\ell -1\right]⋃\left\{v\in V\cup E:|N(v)\cap I[S,\ell -1]|\geq t(e)\right\},$$

*where N(v) denotes the neighborhood of v. The process ends at the first iteration ℓ such that I[S,ℓ−1]=I[S,ℓ]. We denote the final influenced sets as $I_{V}\left[S\right]=I\left[S,\ell -1\right]\cap V$ and $I_{E}\left[S\right]=I_{E}\left[S,\ell -1\right]\cap E$.*

**Figure 2:**
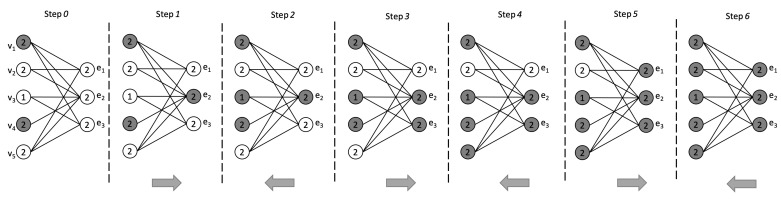
An example of influence diffusion process on I(H) associated with H=(V,E), where $V=\{v_{1},v_{2},v_{3},v_{4},v_{5}\}$ and $E=\{e_{1},e_{2},e_{3}\}$. Influenced nodes appear in gray. The arrow at the bottom of each step indicates the direction of the diffusion process: (left-to-right) nodes influence hyperedges; (right-to-left) hyperedges influence nodes.

A **target set** for H=(V,E) is a seed set S⊆V that will eventually influence the whole set of nodes, i.e., $I_{V}\left[S\right]=V$. It is worth noticing that a target set does not necessarily influence all hyperedges. We indicate the above information diffusion process on I(H), associated with *H*, as

$$I_{V}\left[S\right],I_{E}\left[S\right]=\Phi \left(H,S,t_{V},t_{E}\right),$$

where $I_{V}\left[S\right]\subseteq V$ is the set of influenced nodes and $I_{E}\left[S\right]\subseteq \;E$ is the set of influenced hyperedges. The diffusion process is irreversible; once a node or hyperedge is influenced, it remains in that state until the end of the process. Further, we can note that, being I(H) a bipartite graph, the process proceeds by adding nodes corresponding to the hyperedges of *H* during odd iterations and nodes corresponding to nodes of *H* during even iterations (see Figure 2).

**Example** **1.** *Consider the bipartite incidence graph I(H) associated with the hypergraph H=(V,E) in Figure 1, with node and hyperedge thresholds given in Figure 2. Starting with the initial seed set $S=\{v_{1},v_{4}\}$, the information diffusion process evolves as follows (see Figure 2).*

$Step\;0:\;\;I\left[S,0\right]=S=\left\{v_{1},v_{4}\right\}$

$Step\;1:\;\;I\left[S,1\right]=\left\{v_{1},v_{4},e_{2}\right\}$

$Step\;2:\;\;I\left[S,2\right]=\left\{v_{1},v_{4},e_{2},v_{3}\right\}$

$Step\;3:\;\;I\left[S,3\right]=\left\{v_{1},v_{4},e_{2},v_{3},e_{3}\right\}$

$Step\;4:\;\;I\left[S,4\right]=\left\{v_{1},v_{4},e_{2},v_{3},e_{3},v_{5}\right\}$

$Step\;5:\;\;I\left[S,5\right]=\left\{v_{1},v_{4},e_{2},v_{3},e_{3},v_{5},e_{1}\right\}$

$Step\;6:\;\;I\left[S,5\right]=\left\{v_{1},v_{4},e_{2},v_{3},e_{3},v_{5},e_{1},v_{2}\right\}$

*As $I_{V}\left[S\right]=V$, S is a target set for H.*

In this work, we address the problem of finding the minimum target set that will eventually influence the whole network. Formally, given a hypergraph H=(V,E) with threshold functions $t_{V}:V⟶N$ and $t_{E}:E⟶N$, we aim to find a seed set S⊆V of minimum size such that $I_{V}\left[S\right]=V$.

**Definition** **2.** *TSS problem on hypergraphs (TSSH).*

***Instance:*** *H=(V,E), thresholds $t_{V}:V\to N_{0}$ and $t_{E}:E\to N_{0}$.*

***Problem:*** *Find a seed set S⊆V of minimum size such that $I_{V}\left[S\right]=V$.*

The TSSH problem generalizes the TSS problem on graphs, studied by Chen [33], which is defined as follows:

Given a graph G=(V,E) and fixed arbitrary thresholds t(v), for each v∈V, find a set of minimum size that eventually influences all nodes of *G*.

Recalling that any graph is a hypergraph, TSSH solves TSS by setting $t_{V}\left(v\right)=t\left(v\right)$ for each v∈V and $t_{E}\left(e\right)=1$ for each e∈E. Chen proved a strong inapproximability result on TSS showing that, even a non-trivial approximation of the problem, is unlikely tractable. Being TSSH a generalization of TSS, Chen’s result also applies to TSSH.

It is worth mentioning that having $t_{E}\left(e\right)\geq 1$ on the TSSH problem does not imply that spreading influence is more difficult in hypergraphs than in graphs as the diffusion process is ruled by both the thresholds and the size of the hyperedges. For instance, let us assume that all nodes have a threshold 2 and the existence of two hyperedges with a threshold 2, whose intersection contains 100 nodes. In this case, two influenced nodes in the intersection of the two given hyperedges can influence the other 98 nodes.

## 4. Finding the Minimum Target Set on Hypergraphs

In this section, we present four greedy-based heuristics for the *TSSH* problem defined above; i.e., given a hypergraph H=(V,E) having threshold values described by the functions $t_{V}$ and $t_{E}$, find the minimum target (seed) set S⊆V able to influence the whole network. We indicate the diffusion process on *H* as Φ(H,S), denoting with $I_{V}\left[S\right]\subseteq V$ and $I_{E}\left[S\right]\subseteq E$ the final sets of influenced nodes and influenced hyperedges, respectively.

The first three heuristics described below have been defined and preliminarily evaluated in [17]. In this paper, we further provide (i) a different approach for the TSSH problem, defining a pruning heuristic (SubTSSH) and (ii) an opt` as a refinement of all four proposed heuristics, able to provide significant improvements on the size of the desired target set.

**StaticGreedy.** A trivial greedy strategy consists of selecting the nodes with the highest degree as a seed set. After sorting the nodes in descending order by their degree, the heuristic applies a binary search procedure to compute the target set *S*. This heuristic is referred to as static since the node degree is never updated (see Algorithm StaticGreedy in [17] for a detailed description).

**DynamicGreedy.** In this heuristic, all nodes are initially added to the candidate set *U*. At each iteration, the node having the highest degree is added to the target set *S* and removed from *U*. At this point, the strategy simulates the diffusion process and prunes all influenced nodes and hyperedges from the hypergraph. Then, the residual degree δ(v) of each node *v* is updated accordingly, and the process is iterated until all nodes are influenced or pruned (see Algorithm DynamicGreedy in [17] for a detailed description).

${\mathrm{DynamicGreedy}}_{{\left[H\right]}_{2}}$**.** This third heuristic is equivalent to DynamicGreedy, but it is computed on the weighted clique-expansion ${\left[H\right]}_{2}^{w}$ of the input hypergraph *H*. As a consequence, each node degree is evaluated on ${\left[H\right]}_{2}^{w}$ (see Algorithm $DynamicGreedy_{{\left[H\right]}_{2}}$ in [17] for a detailed description).

**SubTSSH.** This heuristic, which extends the TSS Algorithm in [19], works greedily by iteratively pruning nodes or edges from the input hypergraph *H* unless a specific condition occurs, making a node being added to the seed set *S*; it stops when all nodes have either been discarded or selected as seeds. The pruning is done according to a designed rule that tries to balance between the capability of a node (resp. edge) to influence other edges (resp. nodes) and its “easiness” (or hardness) to be influenced by other edges (resp. nodes). In contrast to the previous three heuristics, which exploit an additive approach, this technique adopts a subtractive procedure.

Algorithm 1, $SubTSSH(H=\left(V,E\right),t_{V},t_{E})$, proceeds as follows. As long as the hypergraph is not empty and no special conditions occur (e.g., cases 1 or 2), a node *u* (case 3.a) or an edge *e* (case 3.b) is selected according to a given function (see Case 3) and it is pruned from the hypergraph. When a node (resp. edge) is removed, its incident edges (resp. nodes) update their size (resp. degree) as they cannot count on *u* (resp. *e*) anymore to become influenced. Due to this update, some nodes in the residual hypergraph may remain with less “usable” edges (if a node u∈V has $\delta \left(u\right)<t_{V}\left(u\right)$). In such a case (see Case 2), these nodes are added to the seed set *S* and removed from *H*. Then, the size and the thresholds of their incident edges are updated (i.e., decreased by 1) since they have one more influenced neighbor. In the last case (see Case 1), the residual hypergraph contains a node *u* (resp. edge *e*) whose threshold decreased down to 0. This situation means that the nodes which have been already added to the seed set *S* are enough to make *u* (resp. *e*) influenced. At this point, *u* (resp. *e*) is removed from *H*, and both the size and the thresholds of its incident edges (resp. nodes) are updated (i.e., decreased by 1) since they have one more influenced neighbor.

**Optimization phase.** Being greedy-based approaches, all the described algorithms ensure that $I_{V}\left[S\right]=V$, but they do not guarantee that the cardinality of *S* is (an approximation of) the optimal solution. Moreover, we experimentally observed that their solutions often contained unnecessary seed nodes. Given a seed set *S* such that $I_{V}\left[S\right]=V$, a node v∈S is unnecessary if $I_{V}\left[S-\left\{v\right\}\right]=V$. Since we are looking for a minimum cardinality solution, it is important to remove all superfluous nodes. For this reason, we developed a skim procedure, named OptimizationStrategy and listed in Algorithm 2, which, starting from a solution obtained by the above algorithms, iteratively removes all redundant nodes.
**Algorithm 1**$SubTSSH(H=\left(V,E\right),t_{V},t_{E})$1:S=∅2:**while**V≠∅**do**3:    **if** $\exists \;u\in V\;|\;t_{V}\left(u\right)=0$ **then**        ▹ Case 1.a: if a node exists with threshold 0, it is self-influenced4:        V=V−{u};         ▹ Remove *u* from the hypergraph5:        UpdateThresholds(H,u);         ▹ Reduce the thresholds of the edges containing *u*6:        UpdateSizes(H,u);         ▹ Reduce the size of the edges containing *u*7:    **else**8:        **if** $\exists \;e\in E\;|\;t_{E}\left(e\right)=0$ **then**        ▹ Case 1.b: if an edge exists with threshold 0, it is self-influenced9:           E=E−{e};         ▹ Remove *e* from the hypergraph10:           UpdateThresholds(H,e);         ▹ Reduce the thresholds of the nodes belonging to *e*11:           UpdateDegrees(H,e);         ▹ Reduce the degree of the nodes belonging to *e*12:        **else**13:           **if** $\exists \;u\in U\;|\;\delta \left(u\right)<t_{V}\left(u\right)$ **then**        ▹ Case 2: *v* cannot be influenced by its neighbors14:               S=S∪{u};         ▹ Add *u* to the seed set *S*15:               V=V−{u};         ▹ Remove *u* from the hypergraph16:               UpdateThresholds(H,u);         ▹ Reduce the thresholds of the edges containing *u*17:               UpdateSizes(H,u);         ▹ Reduce the size of the edges containing *u*18:           **else**        ▹ Remove a node *v* or an edge *e*19:               $u=argmin_{v\in V}\frac{t_{V}\left(v\right)}{\delta (v)(\delta (v)+1)}$         ▹δ(v) is the current degree of the node *v*20:               $e=argmin_{he\in E}\frac{t_{E}\left(he\right)}{\lambda (he)(\lambda (he)+1)}$        ▹λ(he) is the current size of the edge he21:               **if** $\frac{t_{V}\left(u\right)}{\delta (u)(\delta (u)+1)}<\frac{t_{E}\left(e\right)}{\lambda (e)(\lambda (e)+1)}$ **then**        ▹ Case 3.a: Remove *u*22:                   V=V−{u};         ▹ Remove *u* from the hypergraph23:                   UpdateSizes(H,u);         ▹ Reduce the size of the edges containing *u*24:               **else**        ▹ Case 3.b: Remove *e*25:                   E=E−{e};         ▹ Remove *e* from the hypergraph26:                   UpdateDegrees(H,e);         ▹ Reduce the degree of the nodes belonging to *e*27:return *S*


**Algorithm 2**
$OptimizationStrategy(H=\left(V,E\right),t_{V},t_{E},S)$

1:Let $S_{\pi }=\left[s_{1},s_{2},\ldots ,s_{t}\right]$ be a vector with all nodes in *S* in non-increasing order of their degree.2:
**for** i∈[1,t] **do**
3:    $I_{V}\left[S_{\pi }\right],I_{E}\left[S_{\pi }\right]=\Phi \left(H,S_{\pi }-\left\{s_{i}\right\},t_{V},t_{E}\right)$4:    **if** $I_{V}\left[S_{\pi }\right]=V$ **then**5:        $S_{\pi }=S_{\pi }-\left\{s_{i}\right\}$;6:return $S_{\pi }$


## 5. Experiments

To evaluate the effectiveness of the greedy-based heuristics discussed in Section 4, we performed a bunch of experimental scenarios varying the activation threshold for both nodes and hyperedges. Specifically, we assessed their performance on several real-world networks considering both the cardinality of the solution provided (the smaller, the better) and their execution times.

### 5.1. Data Sets

As benchmark hypergraphs, we used 11 networks generated by real-world data sets, downloaded from the ARB [34], Mendeley [35] and GitHub [36] repositories. A more detailed description of each data set follows. Table 1 and Table 2 summarize the dimension of each hypergraph, along with the number of edges in the corresponding clique-expansion. The size shown refers to the final dimension of the hypergraphs after having removed any 0-degree node and empty hyperedges.

- *Algebra*. Nodes are users of mathoverflow.net, and hyperedges correspond to users who answered a particular type of question about algebra within a month.
- *Amazon*. Sets of products reviewed by users on Amazon. Each node corresponds to a product, and a hyperedge links together groups of similar items. We used a subset of reviews.
- *DBLP*. Co-authorship on DBLP papers. Each node is an author, while each hyperedge is a publication. We considered all documents published between January and May 2017.
- *Email-Enron*. Sets of email addresses involving Enron employees. In this data set, nodes are email addresses at Enron, and a hyperedge comprises the sender and all recipients of the email. We considered a subset of emails.
- *Email-W3C*. Sets of email addresses on W3C mailing lists. Each hyperedge consists of a set of email addresses, which have all appeared on the same email. We considered a subset of emails.
- *Geometry*. Hypergraph where nodes are users of mathoverflow.net, and hyperedges are sets of users who answered a certain question category about geometry.
- *Game of Thrones (GoT)*. Hypergraph where nodes are GoT characters, and hyperedges are GoT scenes. In other words, a hyperedge consists of all characters appearing in the same scene together. More details about this data set are given in [37].
- *Blues Music Reviews*. Hypergraph where nodes are Amazon users and hyperedges link users who reviewed a certain product category (different types of blues music) within a month timeframe.
- *NBA*. NBA games in the period 1985–2013. Nodes are players, and a hyperedge connects together all players involved in a match up to 2012.
- *Restaurant Reviews*. Hypergraph where nodes are Yelp users, and hyperedges link users who reviewed an establishment of a particular category (different types of restaurants in Madison, WI) within a month timeframe.
- *Bars Reviews*. Hypergraph where nodes are Yelp users, and hyperedges connect users who reviewed an establishment of a particular category (different types of bars in Las Vegas, NV) within a month timeframe.

All hypergraphs used for the experimental part of this paper are available on a GitHub public repository [38] and on Zenodo [39].

### 5.2. Experiment Formulation

We performed four experimental scenarios. In the first and second scenarios, we fixed each node threshold to a random value between 1 and its degree. In the other two scenarios, each node threshold varies proportionally—from 0.2 to 0.8—to the degree of the node. Concerning the hyperedge thresholds, we fixed each edge threshold to a random value between 1 and its degree in the first and third scenarios. We set each hyperedge activation threshold proportional to its degree scaled of factor 0.5 (majority policy) in the remaining settings. Table 3 summarizes the threshold settings in the four experimental scenarios. We simulated the execution of each heuristic followed by the optimization procedure 50 times per experiment. Then, we repeated the experiment simulating the execution of each heuristic without applying the optimization step to compare the new results with our previous work [17]. All experiments as well as the heuristics are implemented in Julia, exploiting the library SimpleHypergraphs.jl [40]. The Julia code used in the paper is available on a GitHub public repository [38] and on Zenodo [41].

#### 5.2.1. Scenario 1 — Random thresholds

**Figure 3:**
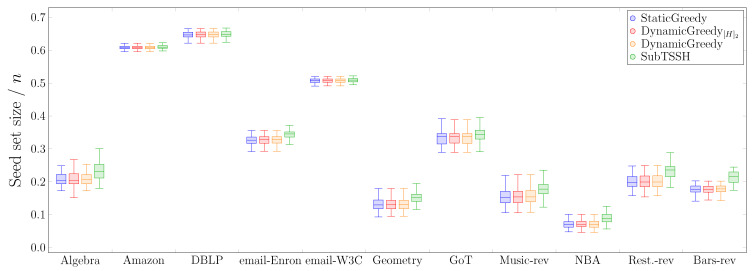
Scenario 1—Ratio of the seed set size over the total number of nodes *n* per hypergraph, fixing each node (hyperedge) threshold to a random value between 1 and its degree (size).

**Figure 4:**
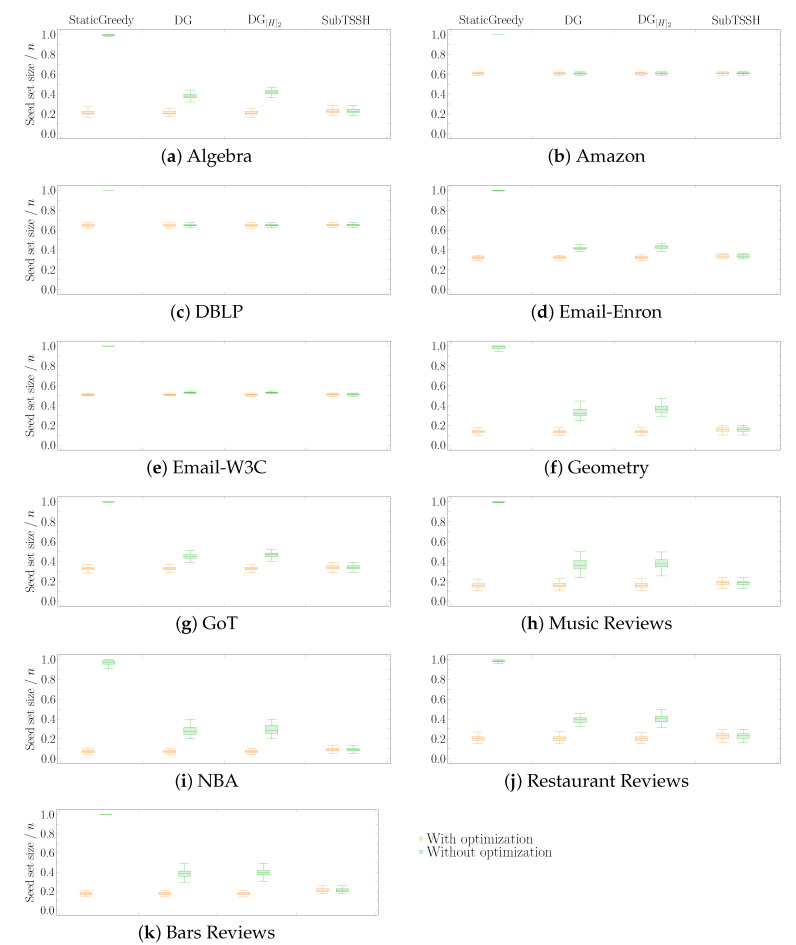
Scenario 1—Ratio of the seed set size over the total number of nodes *n* per hypergraph, fixing each node (hyperedge) threshold to a random value between 1 and its degree (size). The acronyms DG and ${\mathrm{DG}}_{{\left[H\right]}_{2}}$ stand for DynamicGreedy and ${\mathrm{DynamicGreedy}}_{{\left[H\right]}_{2}}$, respectively. Each plot compares the seed set size obtained by running each heuristic with (left—orange) and without (right—green) optimization procedure.

In this first experimental scenario, we fixed each node (hyperedge) threshold to a random value between 1 and its degree (size), varying it at each run of the experiment. Figure 3 shows the distribution of the seed set size per hypergraph using the four heuristics described in Section 4, followed by the optimization procedure. Figure 4 reports the same information zooming into each data set comparing the four heuristics with (left—orange) and without (right—green) the optimization procedure. The size of the seed set is reported as the ratio over the total number of nodes to enable comparison across all data sets.

The first interesting aspect to note is that the optimization phase has a meaningful impact on all three additive heuristics—StaticGreedy, DynamicGreedy, and $DynamicGreedy_{{\left[H\right]}_{2}}$. This behavior is highly emphasized when looking at the StaticGreedy procedure, where the algorithm selects almost the totality of the nodes when run without optimization. Second, the cleaning procedure carried out after selecting the initial seed set leads to achieving very similar results when using the additive heuristics. This outcome may happen because the three heuristics choose a very similar set of core nodes (i.e., the nodes remaining after the optimization phase), even though they initially select different seed sets. On the other hand, the optimization phase has a very low, if not zero, impact on reducing the initial seed set found by the subtractive heuristic SubTSSH. Thus, running the algorithm with and without optimization brings consistent results, always comparable with the outcomes of the additive heuristics. As a result, when using the SubTSSH algorithm, the optimization phase may also be skipped by paying a minimal price on the dimension of the final seed set.

#### 5.2.2. Scenario 2—Random Node Thresholds, Majority Policy on Hyperedges

**Figure 5:**
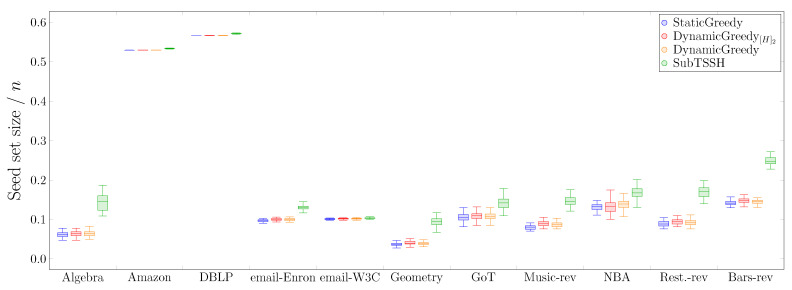
Scenario 2 — Ratio of the seed set size over the total number of nodes *n* per hypergraph, fixing each node threshold to a random value between 1 and its degree and using a majority policy for hyperedge thresholds.

**Figure 6:**
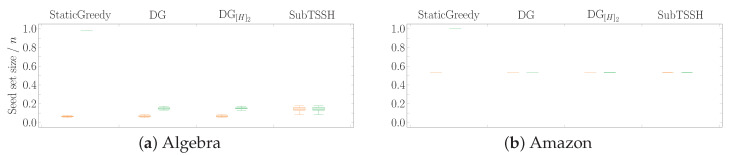

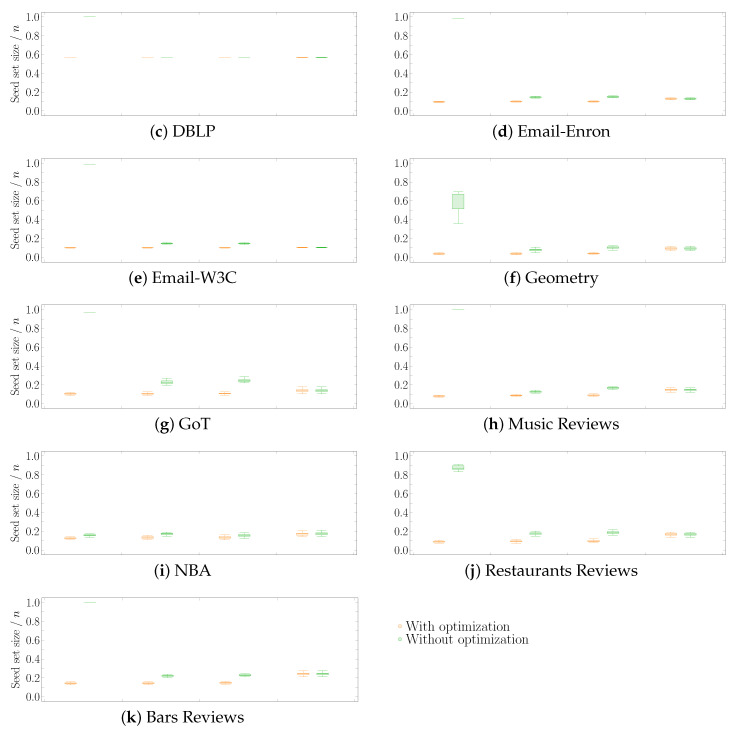
Scenario 2 — Ratio of the seed set size over the total number of nodes *n* per hypergraph, fixing each node threshold to a random value between 1 and its degree and using a majority policy for hyperedge thresholds. The acronyms DG and ${\mathrm{DG}}_{{\left[H\right]}_{2}}$ stand for DynamicGreedy and ${\mathrm{DynamicGreedy}}_{{\left[H\right]}_{2}}$, respectively. Each plot compares the seed set size obtained by running each heuristic with (left—orange) and without (right—green) optimization procedure.

In a second experiment, we fixed each node threshold to a random value between 1 and its degree, varying it at each run of the experiment. We used a majority policy for the hyperedge thresholds instead. Figure 5 shows the distribution of the ratio of the seed set size over the total number of nodes *n* per hypergraph obtained by running the four heuristics with the optimization step. Figure 6 reports the same information zooming into each data set comparing the four heuristics with (left—orange) and without (right—green) the optimization procedure. The results of this experiment resemble the outcomes of *Scenario 1*. As in the previous case, additive heuristics (plus the optimization procedure) perform slightly better than SubTSSH in obtaining the smallest seed set.

Among all data sets, when it comes to select the seed set for the hypergraphs associated with *Amazon* and *DBLP*, all heuristics (with and without optimization procedure) pick at least half of the total number of nodes. This result is emphasized by the smaller number of nodes (in percentage) required to fully influence the other networks. A similar pattern also happens in Scenario 1 (see Figure 3). The reason for this behavior has to be sought in the topology of the networks. What differentiates the *Amazon* and *DBLP* hypergraphs from the others is that their number of nodes is at least 68% higher than their number of hyperedges. In addition, the majority of the nodes of the two networks has degree equal to 1 (97.89% for *Amazon*, 97.21% for *DBLP*). Being involved in only one relation, these nodes are difficult to influence and, at the same time, cannot help in influencing other nodes; thus, confirming how the topology of the network strongly determines the minimum target set needed to influence the whole hypergraph. As expected, StaticGreedy performs the worst when not followed by the cleaning step, selecting in all hypergraphs but *NBA* the highest number of nodes (if not almost all nodes). In line with our previous work [17], this outcome suggests that using a dynamic approach is critical to select a reasonable seed set.

#### 5.2.3. Scenario 3—Proportional Node Thresholds, Random Hyperedge Thresholds

In this third experimental scenario, we varied each node threshold proportionally—from 0.2 to 0.8—to the degree of the node and fixed each hyperedge threshold to a random value between 1 and its size. It is worth stressing that the higher the threshold, the harder it is to influence the given node or hyperedge.

**Figure 7:**
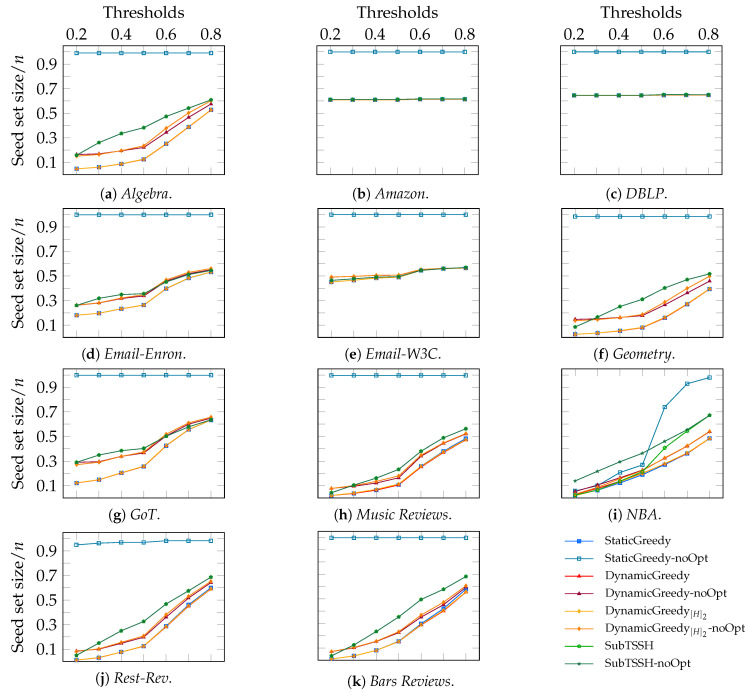
Scenario 3 — Ratio of the seed set size over the total number of nodes *n* per hypergraph, varying each node threshold proportionally - from 0.2 to 0.8 - to its degree, and fixing each hyperedge threshold to a random value between 1 and its size.

Figure 7 shows the ratio of the seed set size over the total number of nodes *n* separately for each hypergraph. These outcomes look like the ones previously discussed in *Scenario 1* and *Scenario 2*. Overall, the additive heuristics followed by the optimization procedure performed better in all data sets, consistently achieving a smaller seed set than SubTSSH, regardless of the node threshold value. Generally, using proportional node thresholds, DynamicGreedy and $DynamicGreedy_{{\left[H\right]}_{2}}$ without the cleaning procedure tend to perform equal or better than SubTSSH (with and without optimization). A plausible explanation for this behavior must be sought in how each algorithm selects the next node to add to the seed set. Specifically, DynamicGreedy and $DynamicGreedy_{{\left[H\right]}_{2}}$ choose the nodes to put in the seed set only based on their degree. In this specific setting, selecting the nodes with a higher degree increases the probability of influencing a higher number of nodes (being each node’s threshold proportional to its degree). On the other hand, SubTSSH prunes from the hypergraph nodes that are easy to influence, even though they may have many neighbors; hence, removing possible good candidates. As already discussed in the previous two scenarios, the StaticGreedy algorithm without the cleaning step always produces a seed containing almost all nodes. Further, as before, optimizing the result of the SubTSSH procedure brings little or no improvements to the seed set size.

Again, this experiment draws attention to the *Amazon* and *DBLP* hypergraphs, as it clearly stands out that all heuristics have the same performance (as also happens in the first two experiments, see Figure 3 and Figure 5). We can further observe the same behavior for the *Email-W3C* hypergraph. As already discussed, the reason for that lies in the number of nodes being part of only one relation (80.57% of the nodes have degree 1).

#### 5.2.4. Scenario 4—Proportional Node Thresholds, Majority Policy on Hyperedges

In the fourth and last experimental scenario, we varied each node threshold proportionally—from 0.2 to 0.8—to the degree of the node, while we used a majority policy for hyperedges.

**Figure 8:**
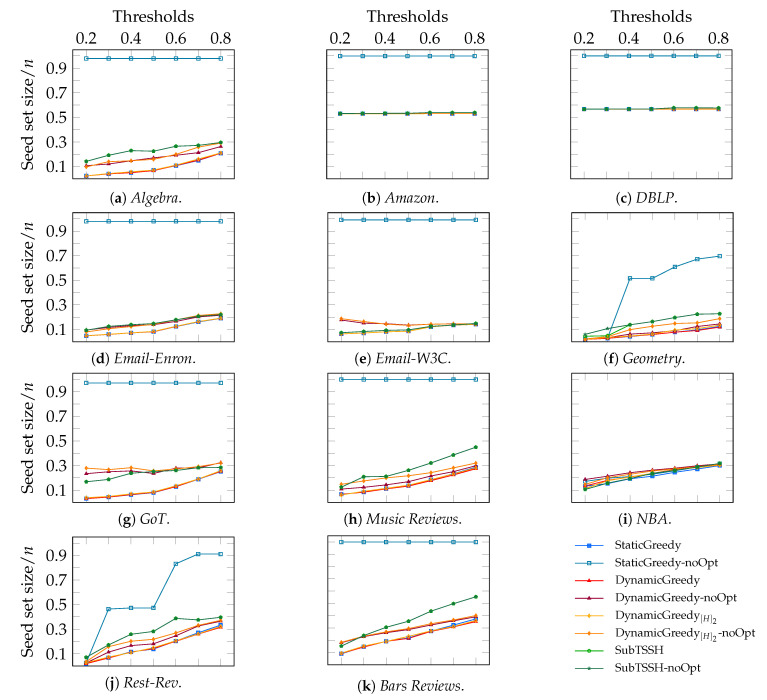
Scenario 4—Ratio of the seed set size over the total number of nodes *n* per hypergraph, varying each node threshold proportionally—from 0.2 to 0.8—to its degree, and using a majority policy for hyperedge thresholds.

Figure 8 shows the ratio of the seed set size over the total number of nodes *n* separately for each hypergraph. We can observe a familiar picture, where the three additive heuristics—followed by the optimization step—retrieve the smallest seed sets and the three particular cases of the *Amazon*, *DBLP*, and *Email-W3C* hyper-networks where all heuristics (but StaticGreedy without optimization) achieve very similar results. Generally, as also observable in Figure 5, fewer nodes are required to influence the whole network when a majority policy on hyperedges is used. This outcome means that, on average, hyperedges with a higher threshold make the problem harder.

### 5.3. Execution Time Comparison

Having investigated the effectiveness of each heuristic in finding the smallest target set able to influence the whole network, we then analyzed their performance in terms of execution time. Specifically, we used the same setting of *Scenario 1*, fixing random thresholds for both nodes and hyperedges. We simulated the execution of each heuristic 50 times, using the Julia package BenchmarkTools.jl. We run each experiment on an Ubuntu 18.04.2 LTS machine, equipped with Intel(R) Xeon(R) CPU E5-2660 0 @ 2.20 GHz and 32 GB RAM.

**Figure 9:**
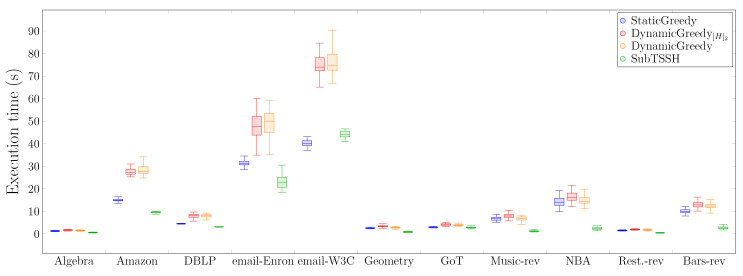
Distribution of the time (in seconds) required for each heuristic to complete.

Figure 9 presents the distribution of the time (in seconds) required for each heuristic to complete the task. The first interesting outcome here, although not surprising, is that additive heuristics consistently need more time than the subtractive technique to accomplish the task. As already discussed in *Scenario 1*, this behavior is due to the fact that additive techniques select many unnecessary nodes demanding a significant workload for the optimization phase, thus increasing the overall time needed to compute the final seed set. As expected, the additive dynamic heuristics—$DynamicGreedy_{{\left[H\right]}_{2}}$ and DynamicGreedy—require the highest computational time, as their algorithms, at each iteration, compute the residual network, updating the nodes candidate set. Generally, the SubTSSH subtractive heuristic requires at least half of the time of the dynamic additive heuristics to complete, thus representing a practical alternative when dealing with bigger hyper-networks.

**Figure 10:**
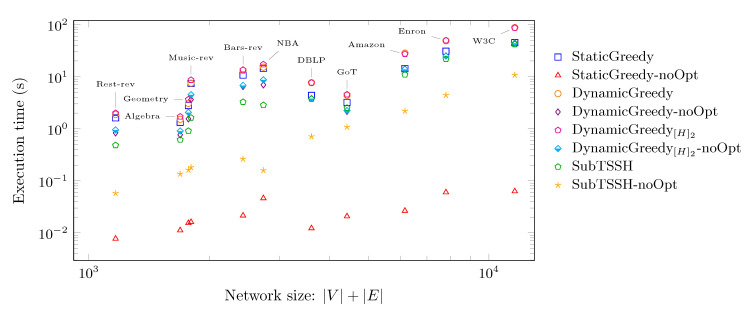
Distribution of the time (in seconds) required for each heuristic to complete the task against the size of the network (|V|+|E|).

**Figure 11:**
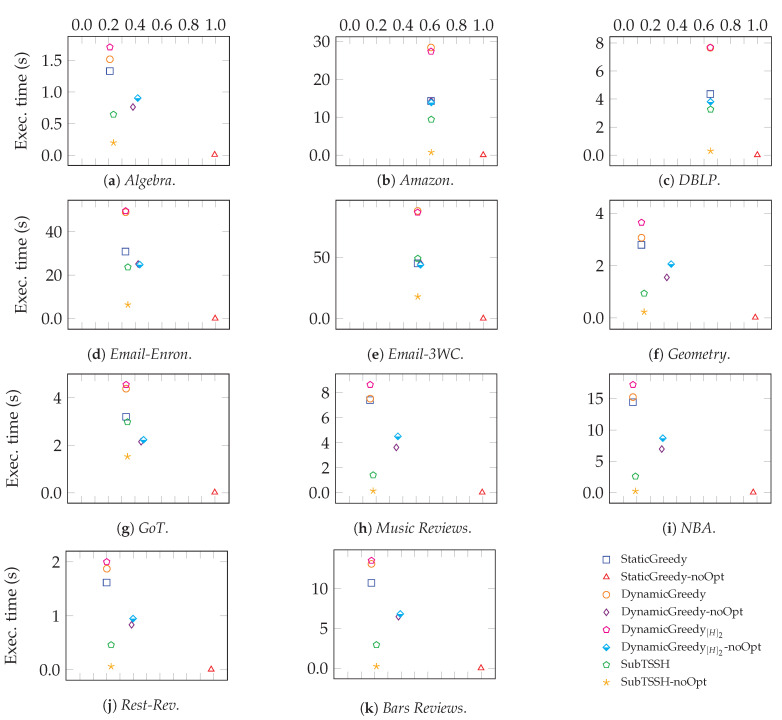
Average execution time vs seed set size.

Figure 10 shows the same results under a different perspective: in this case, the time needed for each heuristic to complete the task is plotted against the network size, evaluated as |V|+|E|. Thus, it allows us to analyze how each algorithm scales with increasing hyper-network size. This time, we also show the results associated with the four greedy strategies—StaticGreedy, $DynamicGreedy_{{\left[H\right]}_{2}}$, DynamicGreedy, and SubTSSH—run without the optimization phase. On the *y*-axis, the average running time of each heuristic is reported. Both axes have a logarithmic scale. Consistently with the data shown in Figure 9, SubTSSH followed by the optimization procedure achieves a very similar or even better performance of all the three additive heuristics run with the optimization phase. The StaticGreedy procedure run without optimization obtains the best time in absolute. However, as shown in Figure 11, it selects the majority of the nodes as seed set; thus, retrieving a useless solution in practice. That leaves SubTSSH executed without the optimization phase as the winner. As already discussed in the experiment of Scenario 1 (see Section 5.2), the optimization phase has a very low, if not zero, impact on reducing the initial seed set evaluated by the SubTSSH heuristic. Hence, the optimization phase may also be skipped when using this algorithm by paying a minimal price on the final seed set’s dimension but still gaining execution time.

Figure 11 zooms into each data set analyzed, comparing the average completion time of each heuristic against the size of the seed set evaluated. As expected, $DynamicGreedy_{{\left[H\right]}_{2}}$ and DynamicGreedy executed without the optimization phase need up to half of the time to complete than their corresponding optimized version. Obviously, the target set evaluated is larger. As previously discussed, the non-optimized version of StaticGreedy confirms the outcomes in [17]: in all networks, this trivial heuristic basically selects all nodes. This result suggests that TSSH heuristics designed for real-world networks should consider both the dynamicity of the influence process and the role that hyperedges play in the whole diffusion mechanism. Further, this experiment clarifies that SubTSSH reaches the best trade-off between the quality of the solution and the time needed to compute it.

## 6. Conclusions

In this paper, we presented a generalization of the linear threshold diffusion model on hypergraphs. The proposed model notably differs from the corresponding model on graphs as it also involves the hyperedges in the diffusion process; thus, enabling the direct modeling of complex group dynamics happening in high-order networks. A challenging problem arising in this domain—known as Target Set Selection (TSS) problem—is to determine the size of the smallest subset of nodes (*a seed-set*) able to spread their influence in the whole network. Based on the previously defined linear threshold diffusion model on hypergraphs, we generalized the TSS problem on such structures (TSSH problem).

To address the TSSH problem, we discussed three greedy-based additive heuristics, improved with respect to the approach proposed in [17] thanks to the inclusion of an additional optimization strategy. We further proposed a subtractive procedure to provide good solutions without the need for an expensive optimization strategy. We evaluated the effectiveness of the proposed heuristics in terms of the cardinality of the solution obtained and their execution time by running extensive experiments on several real networks, varying the activation thresholds for both nodes and hyperedges. Results have shown that, although additive procedures generally provide worse results initially, they particularly benefit from the use of the optimization procedure, whose objective is to remove unnecessary nodes from the solution. This process translates into better results than the subtractive procedure (for which the optimization procedure is practically irrelevant). Results on execution times depicted a completely reversed picture. Although efficient, the optimization procedure has a negative impact on the additive approaches’ running time, thus making those heuristics more time-consuming.

Comparing the seed set size obtained on the different networks, it is possible to observe how generally the results are pretty heterogeneous and strictly depend on the networks’ characteristics, such as the density of the network or the distribution of both node degrees and hyperedge size. In general, it is not easy to foresee what will be the results of a diffusion process on such complex networks.

There are several interesting directions for future work. First, the same procedures discussed in this paper may be exploited to measure influence diffusion over groups (hyperedges) rather than over single individuals (nodes). In real-world or online social networks, groups play an essential role in understanding people’s personal behaviors. Therefore, it is critical to systematically study how the many influences the single person when final decisions depend upon groups of people. Second, motivated by the lack of extensive studies on information diffusion processes on high-order structures, another research path could be developing other diffusion models (e.g., Independent Cascade model) suitable for hypergraphs. Having several approaches to analyze influence diffusion patterns will allow using the most suitable model according to the interactions happening in the real world. Finally, another interesting line of inquiry would be studying diffusion phenomena in heterogeneous hypergraphs, where both nodes and relations may be of more than one type, thus giving birth to different diffusion processes that may influence each other.

The following abbreviations are used in this manuscript:

## Figures and Tables

**Table 1:** Benchmark hypergraphs details (1).

	Algebra	Amazon	DBLP	Email-Enron	Email-W3C	Geometry
|V|	423	4989	2727	2807	5601	580
|E|	1268	1176	874	5000	6000	1193
${\left|E\right|}_{\left[H_{2}\right]}$	50,209	11,590	4298	88,926	11,130	205,127
Source	[34]	[34]	[35]	[34]	[34]	[34]

**Table 2:** Benchmark hypergraphs details (2).

	GoT	Music-Rev	NBA	Restaurants-Rev	Bars-Rev
|V|	577	1106	567	565	1234
|E|	3840	694	2163	601	1194
${\left|E\right|}_{\left[H_{2}\right]}$	23,083	149,288	456,251	31,241	167,000
Source	[36]	[34]	[35]	[34]	[34]

**Table 3:** Threshold settings in the four experimental scenarios.

	Node Thresholds		Hyperedge Thresholds	
	**Random**	**Proportional**	**Random**	**Majority Policy**
**Scenario 1**	*		*	
**Scenario 2**	*			*
**Scenario 3**		*	*	
**Scenario 4**		*		*

## Data Availability

The data sets used for the experimental part of this paper are available on a GitHub public repository [38] and on Zenodo [39].
